# Supplementary material for: Transcriptomic Analysis of Colorectal Cancer Cells Treated with Oil Production Waste Products (OPWPs) Reveals Enrichment of Pathways of Mitochondrial Functionality
Source: Cells. 2022 Dec 10;11(24):3992. doi: 10.3390/cells11243992 (PMC9776412; doi:10.3390/cells11243992)

Transcriptomic analysis of colorectal cancer cells treated with Oil Production Waste Products (OPWPs) reveals enrichment of pathways of mitochondrial functionality

Manuela Leo[§], Livio Muccillo[§], Erica Pranzini, Giovannina Barisciano, Matteo Parri, Giulia Lopatriello, Marco Carlomagno, Alice Santi, Maria Letizia Taddei*, Lina Sabatino*

Content of Supplementary Information

Supplementary Figure S1

Supplementary Figure S2

Supplementary Table S1 (.xls)

Supplementary Figure S1

Upregulated Pathways

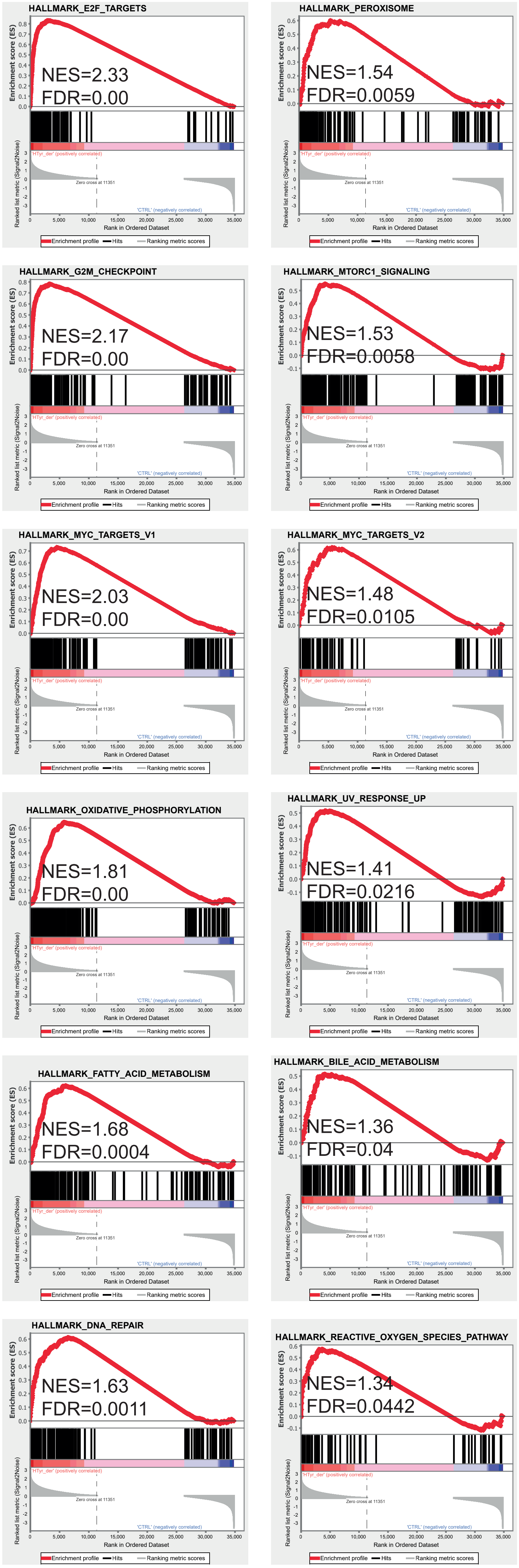

Downregulated Pathways

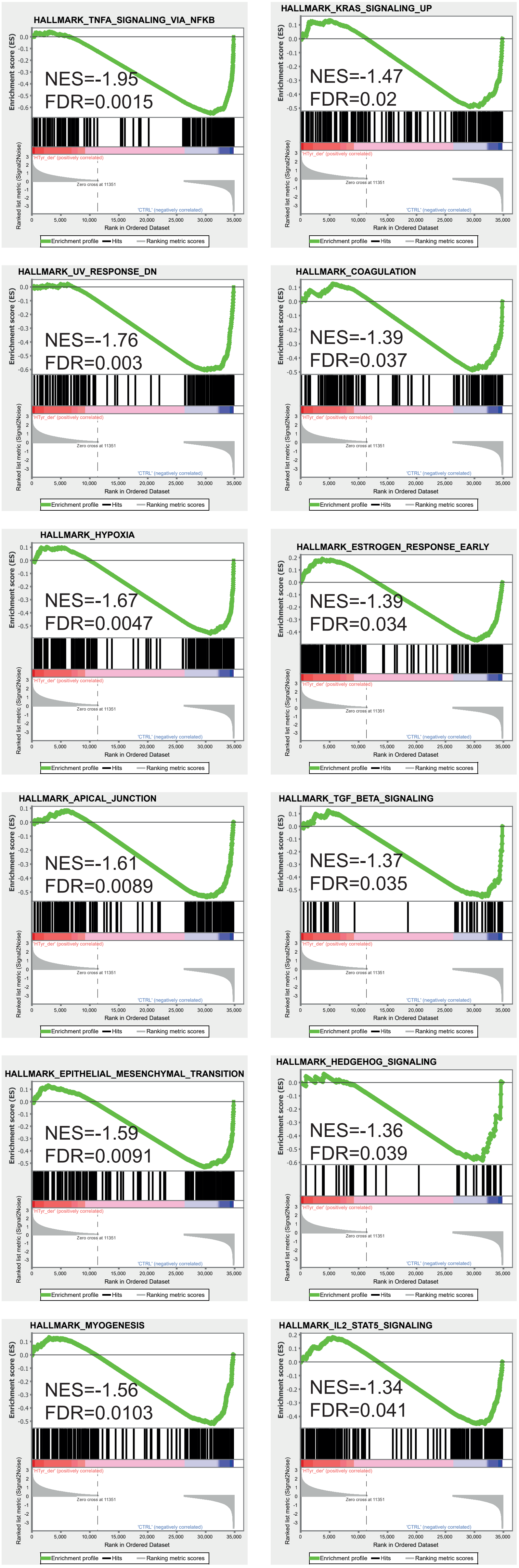

Supplementary Figure S2

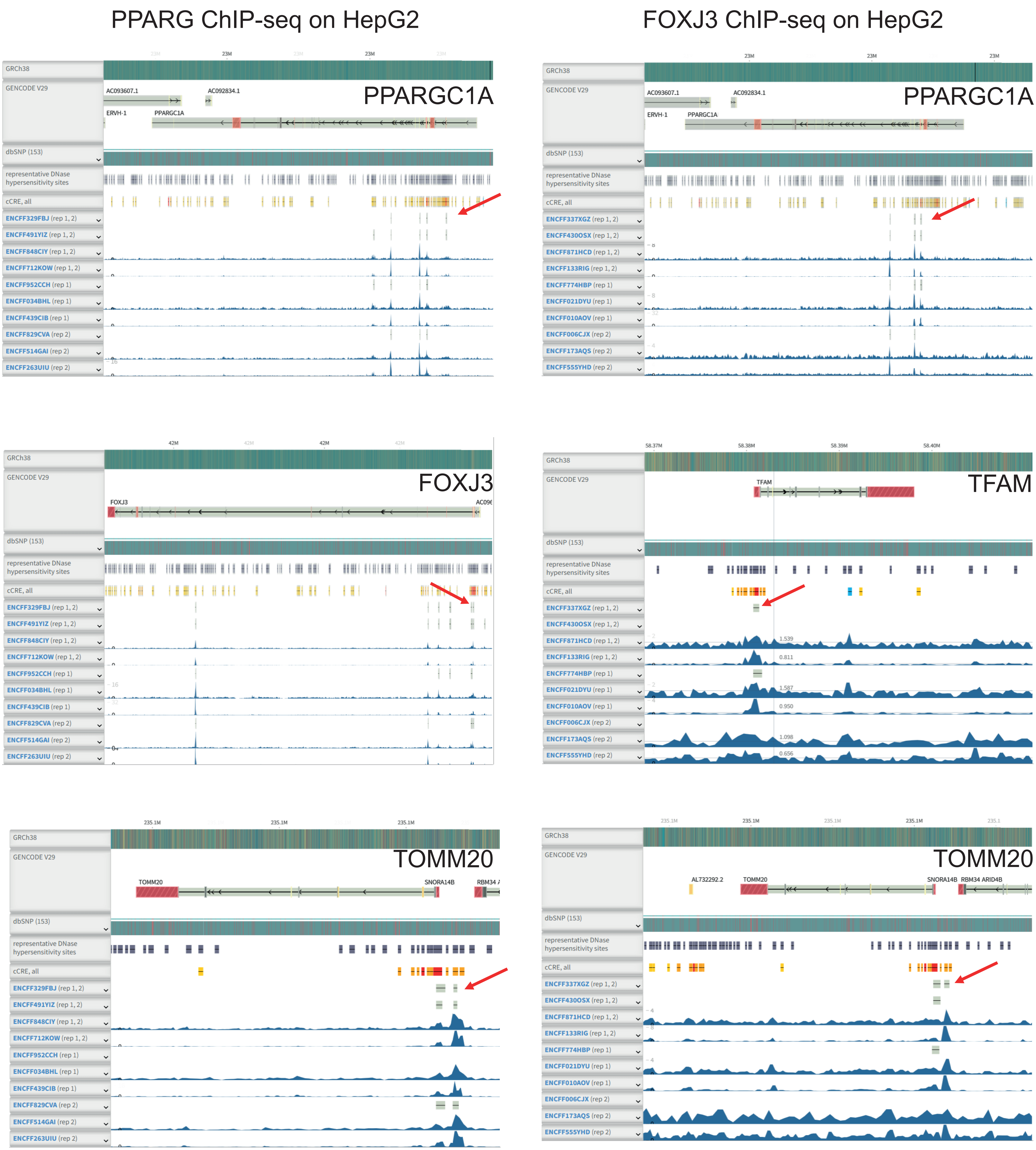

Supplement: Supplementary file 1 [file cells-11-03992-s001.zip › cells-2050540-supplementaryresub.pdf]
